# Impact of Close Margins on Oral Cancer Outcomes According to the Oral Subsite

**DOI:** 10.1002/hed.28024

**Published:** 2024-12-09

**Authors:** Patrick Sheahan, Deirdre Callanan, Nadia van den Berg, Justin Hintze, David Brinkman, Hadeel Jawad, Ryan O'Sullivan, Ross O'Shea, Andrew Dias, Linda Feeley

**Affiliations:** ^1^ Dept of Otolaryngology South Infirmary Victoria University Hospital Cork Ireland; ^2^ Department of Surgery University College Cork Cork Ireland; ^3^ ENTO Research Unit, College of Medicine and Health University College Cork Cork Ireland; ^4^ Department of Pathology Cork University Hospital Cork Ireland

**Keywords:** close, margins, oral cavity, squamous cell carcinoma, tongue

## Abstract

**Background:**

The prognostic significance of close margins in oral squamous cell carcinoma (OSCC) is controversial. We wished to investigate the impact of close margins on the risk of local recurrence (LR) in OSCC according to the oral subsite.

**Methods:**

A retrospective cohort study of 342 OSCC patients undergoing primary surgical treatment was conducted. Surgical margins were based on the main specimen and defined as positive (SCC at margins), close (< 5 mm), or clear (≥ 5 mm).

**Results:**

Among tongue SCC cases, both positive (hazard ratio 13.48, 95% CI 2.03, 32.91) and close margins (hazard ratio 3.87, 95% CI 1.31, 11.34) were significantly associated with LR. Tongue margins < 4 mm were associated with higher LR. Among non‐tongue SCC cases, only positive margins (hazard ratio 4.10, 95% CI 1.19, 14.21) were associated with LR. Close margins were not significant (hazard ratio 1.59, 95% CI 0.46, 5.42).

**Conclusions:**

Close margins appear to have a differential impact on LR in OSCC according to the oral subsite.

## 1 Introduction

Involvement of surgical margins after oral squamous cell carcinoma (SCC) resection is well established as an adverse prognosticator for local recurrence (LR) [1, 2, 3] and an indicator for adjuvant therapy [4]. However, the clinical significance of close margins is more controversial. While some authors have reported surgical margins of < 5 mm to be associated with increased risk of LR [5, 6], others have not found such an association, except for tumors with very close (< 1 mm) margins [3, 7, 8, 9]. In addition, it is controversial whether close surgical margins should be considered an indicator for postoperative radiotherapy (PORT), with some authors reporting an improved outcome with PORT in this cohort [5], and others not finding a benefit [10, 11, 12].

Possible reasons for variable results of studies examining the impact of close surgical margins in oral SCC (OSCC) may include variable definitions of close margins, variation in intraoperative margin assessment and control, differing indications for adjuvant therapy, as well as heterogeneity between patient cohorts. An important source of heterogeneity may be subsite distribution. The most common site of OSCC in most Western countries is tongue; however, subsite distribution and proportion of tongue SCC cases vary between cohorts. SCC at different oral subsites may show differences in risk factors, clinicopathological features at presentation, and prognosis [13]. It is generally accepted that the size of a safe surgical margin may differ across different head and neck sites [14]. However, it is not known whether the primary tumor subsite within the oral cavity has any bearing on the impact of close margins.

In the present study, we hypothesize that close surgical margins may have a differential impact on the risk of LR of OSCC according to the tongue subsite versus non‐tongue subsite.

## 2 Methods

The present study was a retrospective review of a database of patients with OSCC treated at the South Infirmary Victoria University Hospital, which serves as a regional center for Head and Neck Cancer, between 2007 and 2022 inclusive. Included were new cases of OSCC, undergoing primary surgical treatment, with no history of previous Head and Neck Cancer or radiotherapy. Exclusion criteria were non‐squamous histology, recurrent tumors, second primary Head and Neck Cancer, distant metastatic disease, or with no follow‐up available. Permission to perform the study was granted by the Cork Clinical Research Ethics Committee.

Included patients were identified by review of a prospectively maintained Head & Neck Cancer database. Clinical outcomes were established from review of the database and patient notes. A database containing pathological details of all patients with oral SCC was created by extraction of data from pathology reports, with re‐review of original slides in case of missing information. In addition, slides of all cases prior to 2017 were re‐reviewed for remeasurement of depth of invasion according to the 8th edition AJCC TNM staging manual. For later cases, reporting was performed according to the updated 8th edition staging.

Surgical management of OCSCC generally encompassed wide en‐bloc resection of the primary tumor, with the intention of achieving a gross 10 mm 3‐dimensional clearance. Intraoperative frozen sections were used selectively and generally consisted of tumor bed sampling but were not incorporated into margin definition or measurements. Neck dissections were performed as appropriate. Recommendations for PORT were made after discussion of surgical pathology at the Head and Neck multidisciplinary meeting. General indications for PORT included pathological nodal metastases, extranodal extension (ENE), large tumors (T3/4, or > 10 mm depth of invasion), or involved or very close (< 1 mm) margins. Relative indications included perineural invasion (PNI). For the purpose of the study, all patients commencing prescribed PORT were considered to have received the same, regardless of whether they had completed the course.

### 2.1 Definitions

OSCC cases were divided into tongue and non‐tongue subsites. The closest distance on the main specimen between the inked edge and invasive cancer was recorded in millimeters. In all cases, margins were defined on the main resection specimen and did not incorporate results of extra frozen sections or tumor bed resections. Positive margins were defined as invasive SCC or high‐grade dysplasia at the inked margin of the main specimen. Close margins were defined as closest invasive cancer < 5 mm from the inked edge of the main specimen; and clear margins as closest invasive cancer ≥ 5 mm from the inked edge. Cases with negative mucosal/soft tissue margins but positive bone margins were considered positive margin cases.

Staging was performed according to the 8th edition of AJCC/UICC system. LR was defined as recurrent SCC in the same or contiguous oral subsite, irrespective of the elapsed interval since surgery. The second primary tumor (SPT) was defined as new SCC occurring in a non‐contiguous oral or oropharyngeal subsite. Local recurrence free survival (LRFS) was defined as the time interval between surgery and the date of LR. Patients developing SPTs were censored at the date of diagnosis with SPT.

### 2.2 Statistical Analysis

Statistical analysis was performed using XLSTAT (Addinsoft, France, version 2015.1.03). Comparisons on 2 × 2 contingency tables were performed using Fisher's exact test. Comparisons of normally distributed data were performed using Student's *t*‐test. Survival curves were visualized using the Kaplan–Meier method. Univariate hazard ratios (HR) were calculated using Cox proportional hazards modeling. Multivariate analysis was performed including variables with *p* < 0.1 on univariate analysis, along with postoperative RT and margin status.

## 3 Results

Three hundred and forty‐two patients were included in the study. Table 1 shows clinical and histological features of the study population. 140 (40.9%) patients had tongue SCC, and 202 (59.1%) had non‐tongue SCC. The most common site for non‐tongue SCC was floor of mouth (FOM) (98, 28.7% of all OSCC). Patients with non‐tongue SCC were older than patients with tongue SCC (65.1 vs. 60.9 years, *p* = 0.002), more likely to be smokers (80.1% vs. 68.6% for ever smoking, *p* = 0.01), and had a higher incidence of T3/T4 staged primary tumors (50% vs. 30%, *p* = 0.003) and ENE (19.3% vs. 10.7%, *p* = 0.04). Patients with tongue SCC had a higher incidence of lymphovascular invasion (LVI) (28.7% vs. 18.6%, *p* = 0.04).

**TABLE 1:** Clinicopathological and demographic details of the study population.

		Tongue (*n* = 140)	Non‐tongue (*n* = 202)	*p*
Sex	Male	91 (65%)	134 (66.3%)	
	Female	49 (35%)	68 (33.7%)	0.82
Age (years)		60.9 ± 13.4	65.1 ± 12.5	0.003
Oral subsite	Tongue	140		
	Floor of mouth		98 (48.5%)	
	Lower alveolus		25 (12.4%)	
	Retromolar trigone		27 (13.4%)	
	Buccal mucosa/gingivobuccal sulcus		26 (12.9%)	
	Upper alveolus/palate		14 (6.9%)	
	Lip		12 (5.9%)	
Smokers	Current	51 (36.4%)	109 (54.0%)	0.001 (current smokers vs. non)
	Ex‐smokers	45 (32.1%)	54 (26.7%)	
	Never	44 (31.4%)	39 (19.3%)	0.01 (ever vs. never smokers
Surgery performed	Wide excision + neck dissection	108 (25 bilateral) (77.1%)	161 (58 bilateral) (79.7%)	
	Wide excision alone	32 (22.9%)	41 (20.3%)	0.59
Adjuvant treatment	No adjuvant treatment	72 (51.4%)	98 (48.5%)	> 0.99
	Radiotherapy	55 (39.3%)	87 (43.1%)	
	Chemoradiotherapy	13 (9.3%)	17 (8.4%)	
T‐stage	T1	45 (32.1%)	40 (19.8%)	
	T2	53 (37.9%)	61 (30.2%)	
	T3	28 (20.0%)	27 (13.4%)	
	T4	14 (10%)	74 (36.6%)	*p* = 0.0002 (T1/2 vs. T3/4)
N‐classification	pNx	32 (22.9%)	41 (20.3%)	0.59
	pN0	63 (45.0%)	86 (42.6%)	0.45 (N+ vs. N0) excluding pNx
	pN1	21 (15.0%)	18 (8.9%)	
	pN2a	3 (2.1%)	6 (3.0%)	
	pN2b	8 (5.7%)	13 (6.4%)	
	pN2c	0 (0%)	5 (2.5%)	
	pN3b	13 (9.3%)	33 (16.3%)	0.08
Extranodal extension (ENE)	Present	15 (10.7%)	39 (19.3%)	0.04
Margins	Tumor at margin	6 (deep 5, mucosal 1) (4.3%)	45 (deep 32, mucosal 23, 3 bony only) (22.3%)	< 0.0001
	CAP positive margin	10 (7.1%)	51 (25.2%)	< 0.0001
	Close	80 (57.1%)	122 (60.4%)	0.57
	Clear	54 (38.6%)	35 (17.3%)	< 0.0001
Perineural invasion	Present	51 (36.4%)	80 (39.6%)	0.57
Lymphovascular invasion	Present	26 (18.6%)	58 (28.7%)	0.04
Non‐cohesive invasive front	Non‐cohesive	71 (50.7%)	93 (46.0%)	0.44

Fifty‐eight patients (10 tongue and 48 non‐tongue) had positive margins. This included 48 patients (6 tongue and 42 non‐tongue) with invasive cancer at mucosal/soft tissue margins, 7 (4 tongue and 3 non‐tongue) with high grade dysplasia at margins, and 3 (all non‐tongue) with negative mucosal/soft tissue margins but positive bony margins. Primary sites of cases with positive margins among the non‐tongue group were FOM (23), lower alveolus (9), buccal mucosa (6), hard palate/upper alveolus (6), and retromolar trigone (4). Of note, 3/10 tongue SCC patients with positive margins and 10/15 tongue SCC patients with < 1 mm margins had frozen sections and/or additional tumor bed resections which were reported as negative for malignancy. One further tongue patient with < 1 mm margins underwent revision of margins at a secondary operation. The other patients did not undergo additional sampling. Among non‐tongue cases, 25/48 with positive margins and 15/26 patients with < 1 mm margins underwent additional sampling, which yielded final negative margins in 18 and 14 cases, respectively.

Patients with non‐tongue SCC had a significantly higher incidence of positive margins than tongue SCC patients (48/202 vs. 10/140, *p* < 0.0001) and a significantly lower incidence of clear (> 5 mm) margins (35/202 vs. 54/140, *p* < 0.0001).

One hundred and seventy‐two patients (68 tongue, 104 non‐tongue) received adjuvant treatment. This included 8/10 tongue SCC patients with positive margins, 9/15 tongue SCC patients with < 1 mm margins, 29/48 non‐tongue SCC patients with positive margins, and 16/26 non‐tongue SCC patients with < 1 mm margins. Of note, 4/24 tongue SCC patients, and 8/33 non‐tongue SCC patients, with close (1–5 mm) margins, but without other indications for adjuvant treatment (*p*T1/2 N0, ≤ 10 mm depth of invasion, no PNI), received PORT.

The mean (median) follow‐up was 55 (41) months, and the mean (median) time to censoring for local control was 49 (35) months. LR occurred in 62 (18.1%) patients (27 tongue, 35 non‐tongue). Thirty (8.8%) patients (11 tongue, 19 non‐tongue) developed SPT. Seventy‐eight (27 tongue, 51 non‐tongue) patients died from oral cancer, and 101 (29 tongue, 72 non‐tongue) died from other causes.

**FIGURE 1:**
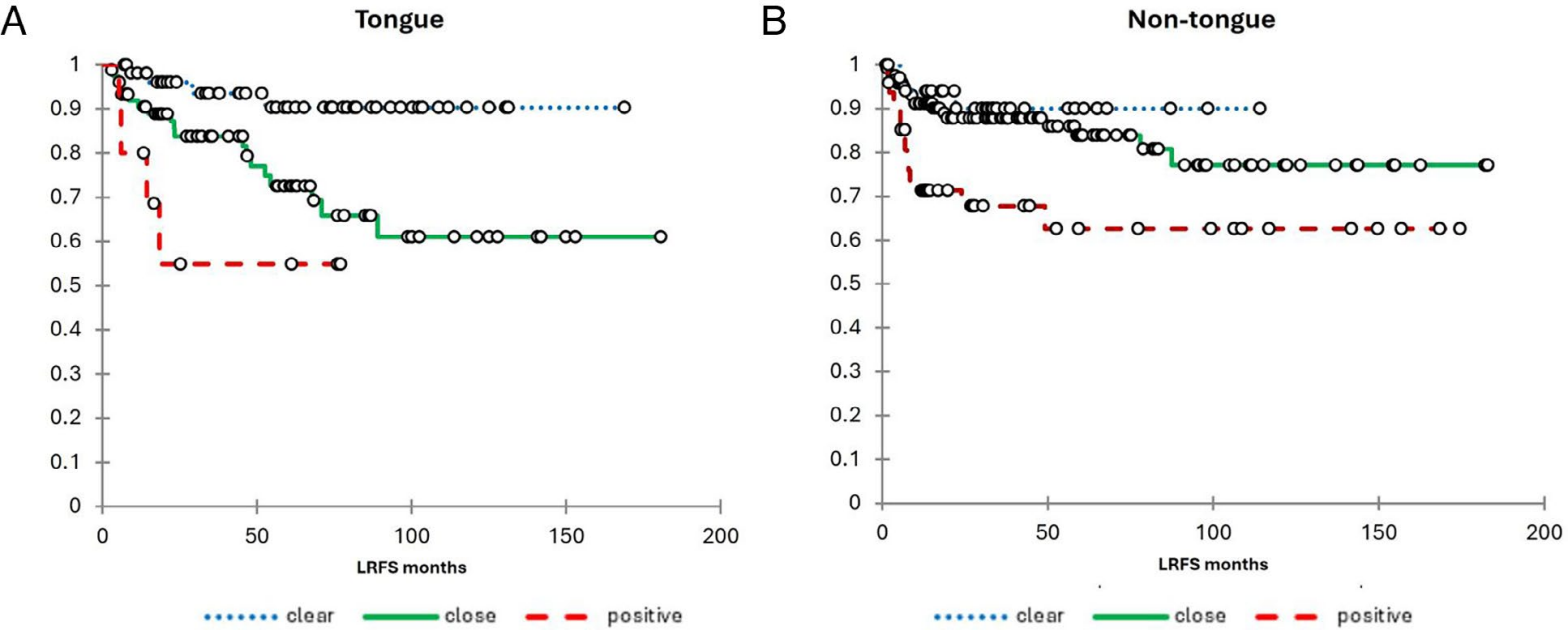
Kaplan Meier curve for local recurrence according to clear, close, and positive margins among patients with tongue (A) and non‐tongue (B) SCC. [Color figure can be viewed at wileyonlinelibrary.com]

Among patients with tongue SCC, there was clear separation of survival curves for LRFS between those with positive, close, and clear margins (Figure 1A). Both positive (hazard ratio 13.48, 95% CI 2.03, 32.91) and close margins (hazard ratio 3.87, 95% CI 1.31, 11.34) were significantly associated with increased risk of LR compared to clear margins. The distance of closest margin as a continuous variable was also significantly associated with LR (*p* = 0.001).

Other factors associated with LR among tongue SCC patients were T‐classification, ENE, and PORT (Table 2). On multivariate analysis, both positive margins (hazard ratio 6.72, 95% CI 1.39, 32.45) and close margins (hazard ratio 3.59, 95% CI 1.17, 11.00) remained independent predictors of LR.

**TABLE 2:** Univariate and multivariate analysis of factors associated with LR in Tongue SCC.

		Univariate hazard ratio (95% CI)	*p*	Multivariate hazard ratio (95% CI)	*p*
Non‐cohesive invasive front		1.53 (0.71, 3.27)	0.27	Not entered	
Perineural invasion		2.12 (0.99, 4.52)	0.05	1.63 (0.66, 4.08)	0.29
Lymphovascular invasion		1.72 (0.73, 4.07)	0.22	Not entered	
T3/4 classification		2.55 (1.17, 5.56)	**0.02**	0.87 (0.31, 2.40)	0.78
pN+ status		1.97 (0.91, 4.26)	0.09	0.88 (0.30, 2.60)	0.82
Extranodal extension		3.96 (1.57, 10.01)	**0.004**	1.57 (0.39, 6.29)	0.52
Margins	Involved	13.48 (2.03, 32.91)	**0.003**	6.72 (1.39, 32.45)	**0.02**
	Close	3.87 (1.31, 11.34)	**0.01**	3.59 (1.17, 11.00)	**0.03**
	Clear	Reference		Reference	
Postoperative radiotherapy		3.03 (1.32, 6.93)	**0.009**	2.19 (0.78, 6.12)	0.14

*Note*: The bold values indicate a significance of *p* < 0.05.

Kaplan–Meier curves for LRFS among patients with non‐tongue SCC are shown in Figure 1B. Positive margins (hazard ratio 4.10, 95% CI 1.19, 14.21) were significantly associated with LR; however, close margins were not significant (hazard ratio 1.59, 95% CI 0.46, 5.42). Other factors associated with LR among non‐tongue cases were T‐classification and PNI (Table 3). On multivariate analysis, positive margins (hazard ratio 3.85, 95% CI 1.09, 13.62) remained significant for LR, along with PNI (hazard ratio 2.63, 95% CI 1.32, 5.23), T‐classification (hazard ratio 3.15, 95% CI 1.43, 6.95), and PORT (hazard ratio 0.33, 95% CI 0.16, 0.69). Close margins were not significant (hazard ratio 1.83, 95% CI 0.53, 6.24).

**TABLE 3:** Univariate and multivariate analysis of factors associated with LR in non‐tongue SCC.

		Univariate hazard ratio (95% CI)	*p*	Multivariate hazard ratio (95% CI)	*p*
Non‐cohesive invasive front		1.27 (0.66, 2.47)	0.48	Not entered	
Perineural invasion		2.86 (1.46, 5.59)	**0.002**	2.63 (1.32, 5.23)	**0.006**
Lymphovascular invasion		1.63 (0.67, 3.97)	0.28	Not entered	
T3/4 classification		2.82 (1.38, 5.80)	**0.005**	3.15 (1.43, 6.95)	**0.004**
pN+ status		1.35 (0.68, 2.66)	0.39	Not entered	
Extranodal extension		1.43 (0.62, 3.30)	0.40	Not entered	
Close margin – 5 mm cutoff	**Involved**	4.10 (1.19, 14.21)	**0.03**	3.85 (1.09, 13.62)	**0.04**
	**Close**	1.59 (0.46, 5.42)	0.46	1.38 (0.53, 6.24)	0.36
Postoperative radiotherapy		0.70 (0.36, 1.36)	0.29	0.33 (0.16, 0.69)	**0.003**

*Note*: The bold values indicate a significance of *p* < 0.05.

Table 4 and Figure S1 show the impact of each 1 mm width of minimum margin clearance on LR. For tongue cases, positive margins (0 mm), 1 mm to < 2 mm margins, 2 mm to < 3 mm margins, and 3 mm to < 4 mm margins, were all associated with significantly increased risk of LR compared to clear (≥ 5 mm) margins, while 0.1 mm to < 1 mm margins were just outside significance (*p* = 0.06). There was no difference in the risk of LR among patients with 4 mm to < 5 mm margins versus clear margins. For non‐tongue cases, only the tumor at margins (0 mm) was associated with an increased risk of LR. No other cut‐off distance had any significant impact.

**TABLE 4:** Impact of each 1 mm incremental margin clearance on the risk of local recurrence for tongue and non‐tongue SCC.

Subsite	Clearance	Number of local recurrences	Hazard ratio (95% CI)	*p*
Tongue	0 mm	4/10	8.25 (2.05, 33.28)	0.003
	0.1 mm to < 1 mm	4/15	3.88 (0.97, 15.52)	0.06
	1 mm to < 2 mm	4/9	7.29 (1.82, 29.31)	0.005
	2 mm to < 3 mm	6/19	6.30 (1.77, 22.38)	0.004
	3 mm to < 4 mm	4/13	4.07 (1.02, 16.30)	0.05
	4 mm to < 5 mm	1/20	0.71 (0.08, 6.39)	0.09
	≥ 5 mm	4/54	Reference	
Non‐tongue	0 mm	15/48	4.10 (1.19, 14.23)	0.03
	0.1 mm to < 1 mm	2/26	0.79 (0.13, 4.73)	0.80
	1 mm to < 2 mm	5/30	1.93 (0.46, 8.08)	0.46
	2 mm to < 3 mm	3/25	1.37 (0.28, 6.82)	0.70
	3 mm to < 4 mm	3/20	1.57 (0.32, 7.77)	0.58
	4 mm to < 5 mm	4/18	2.66 (0.59, 11.91)	0.20
	≥ 5 mm	3/35	Reference	

### 3.1 Impact of Subsites on Margins Within the Non‐Tongue Group

**FIGURE 2:**
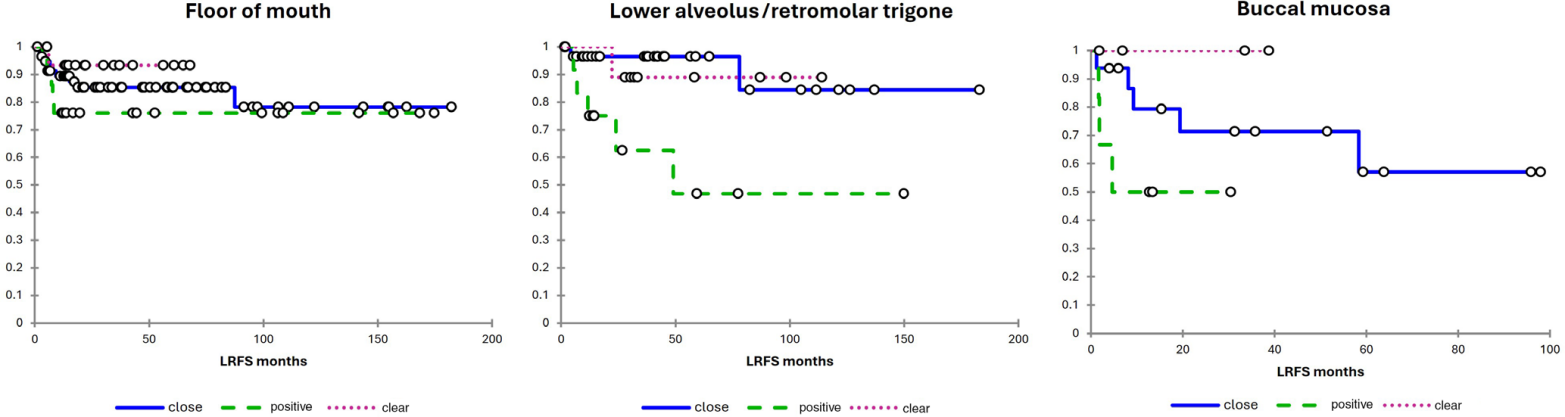
Kaplan Meier curve for local recurrence according to clear, close, and positive margins among patients with SCC of floor of mouth (A), lower alveolus/retromolar trigone (B), and buccal mucosa (C). [Color figure can be viewed at wileyonlinelibrary.com]

Figure 2 shows the Kapan‐Meier survival curves according to the margin status among cases of FOM SCC, lower alveolus/retromolar trigone SCC, and buccal SCC. Clear separation of survival curves between close and clear margins was seen only for buccal SCC; however, the *p*‐value of the log‐rank test was not significant.

### 3.2 Survival

Survival statistics are shown in Tables S1–S4. For tongue cancers, the margin status did not significantly predict survival. For non‐tongue cancers, positive margins were associated with DSS but not OS. Close margins were not associated with either DSS or OS in non‐tongue cases.

## 4 Discussion

In the present study, we hypothesized that the tumor subsite within the oral cavity may influence the prognostic impact of close margins. We found close surgical margins to be a significant adverse prognosticator for LR among tongue cancers, but not for non‐tongue cancers, supporting this hypothesis. To our knowledge, this is the only study looking specifically at the differential impact of close margins on oncological outcomes according to the oral subsite.

Putative reasons for the differential impact of close margins on outcomes of tongue versus non‐tongue SCC include differences in the underlying tissues, such as lack of significant anatomic barriers to tumor invasion within the tongue [15], possible higher frequency of satellite nodules or foci of extratumoral LVI or PNI among tongue cancers [16], or differences in the tumor microenvironment [15]. Of note, in the present series, we did note a higher incidence of LVI among the tongue SCC cases. Another possibility is that tongue cancers are more surgically accessible, and the deep aspect easier to assess by palpation, and therefore it may be easier to ensure wider gross margins. In such cases, if cancer is found unexpectedly close to the resection margin, this may reflect adverse tumor biology with inherent increased risk of LR. On the other hand, in case of cancers of the FOM or other less accessible subsites, a close margin may reflect technical difficulty rather than adverse biology, and may not necessarily portend increased risk of LR, provided the tumor is completely removed. Technical difficulties with resection of non‐tongue cancers may also lead to increased risk of artefactually close margins due to laceration or disruption of the specimen during excision.

In the present series, we also found positive surgical margins to be significantly more frequent among non‐tongue than tongue cancers. This may be partly related to a higher proportion of locally advanced primary tumors in the non‐tongue group, but probably also reflects increased anatomic complexity and technical difficulties in resection of non‐tongue than tongue cancers.

Previous research examining optimum margin cut‐offs in OSCC has reported various distances between 2 mm and 4 mm to be more prognostic than the traditional 5 mm cut‐off [8, 11, 17, 18, 19, 20]. Other studies have reported increased risk of LR only with margins < 1 mm [3, 7]. It is notable that most series that did not find an impact of close margins on recurrence in OSCC contained a large proportion of non‐tongue cases [3, 7, 21], and so it is speculative whether subsite distribution may be a reason for failing to find an impact for close margins in those studies. Besides subsite distribution, other reasons for discrepant results may include variability in the definition and calculation of margins. For example, all included studies in the meta‐analysis of Anderson et al., which reported increased LR with < 5 mm margins, included involved margins together with close margins, so likely biasing the impact of close margins alone [6]. In the paper by Fridman et al., which reported improved local control for PORT among patients with close margins, extra tumor bed resections were incorporated into margin calculations [5]. Thus it is possible that the close margin group included patients with positive margins on the main specimen, which represents a worse prognosis group than patients with negative main specimen margins, and therefore may not be generalizable to margin assessments based on the main specimen alone [22, 23, 24]. Further reasons for the discrepancy in the literature may include variable indications for adjuvant therapy, differences in patient cohorts, and differences in stage distribution [25].

Although the findings of our study would suggest that close margins are an adverse prognosticator in tongue SCC, the optimum cut‐off distance for safe surgical margins remains undefined. The data from the present study would suggest that 4 mm may represent an optimum safe margin. Among series including exclusively patients with tongue SCC, Zanoni et al. reported an optimum margin cut‐off of 2.3 mm (86% with T1/2 disease) [8], while Otsuru et al. in a multicenter study of stage I/II patients reported an optimum cut‐off of 3.3mm [20]. In contrast, Singh et al. reported an optimum cut‐off of 7.6 mm among a series of 451 patients but with more advanced disease (59% stage III/IV) [26].

In contrast, close margins were not found in the present study to be associated with increased risk of LR among non‐tongue cases. However, this finding should be interpreted with caution, as the non‐tongue cohort was a heterogenous group of multiple subsites, and the impact of close margins may not be homogenous within this group. For example, inspection of the Kaplan–Meier curve for buccal cancers would suggest an adverse impact of close margins for this subsite, but the number of cases was too small to detect any significant difference.

An important strength of our study is that we calculated margins based on the main specimen, and not incorporating results of frozen sections and extra tumor bed resections. This is based on previous data showing margin determination from the main specimen to be more prognostic than the final margin status incorporating frozen sections or extra resections [2, 7, 22], and also because frozen sections were only used selectively in this series. It was slightly unexpected in the context of close margins being a significant predictor for LR among tongue SCC cases that the association of margins between 0.1 and < 1 mm and LR was outside significance. However, 11/15 tongue SCC cases with margins between 0.1 and < 1 mm had final negative margins based on additional resections. It is possible that this may have biased results for this subgroup.

An important clinical question is the role of PORT for close margins. We found PORT to have an independent LR benefit among non‐tongue cancers and an OS benefit for both tongue and non‐tongue cancers. However, we cannot rule out confounding due to patients with poor performance status not being offered RT despite risk factors. Notably, PORT was associated with an increased risk of LR among patients with tongue cancers on univariate analysis, which is most likely explained by patients with other adverse prognosticators more likely to be offered PORT. Furthermore, there may have been differences between the tongue and non‐tongue cohorts in this regard due to the younger mean age of patients with tongue cancers.

There are some limitations to our study. These include the retrospective nature and the differences between the tongue and non‐tongue groups. There may also be arbitrariness in some cases regarding the distinction between tongue and FOM cancers. A further important limitation is that the non‐tongue cohort comprises a heterogeneous group of subsites, and therefore our findings may not be automatically generalizable to all non‐tongue subsites. In particular, previous authors have reported an adverse impact of close margins on outcomes among buccal cancers [27, 28], and inspection of the Kaplan–Meier curves of buccal cases in the present series would also suggest an adverse impact for close margins, albeit the number of cases was too small to show a significant impact. Given the small number of buccal SCC cases, we felt it was more appropriate to include them as non‐tongue cases in the present series than to selectively exclude them; however, our findings may not be generalizable to this subsite. Another point is that while it was a strength of our study that margins were calculated based on the main specimen, our findings may not be generalizable to cases where final margin clearance is calculated including extensions to the resection indicated by intraoperative specimen‐driven margin assessment [29, 30, 31]. Finally, it is possible that our study was underpowered to detect a significant difference for close margins on LR of non‐tongue cancers. However, our purpose was to examine whether close margins in OSCC have a differential impact on LR based on the subsite, and, given that we showed a clear impact on LR for close margins with tongue cancer in a smaller cohort than the non‐tongue cohort, this suggests at least a more significant impact of close margins on outcomes among tongue than non‐tongue sites.

## 5 Conclusion

In the present study, we found positive margins to have adverse prognostic impact among both tongue and non‐tongue SCC cases. On the other hand, close margins were associated with LR only among tongue SCC cases. Our findings suggest a differential impact of close margins on outcomes in OSCC based on the primary tumor subsite. More work is required to define a safe margin distance in tongue and non‐tongue sites, as well as define the impact of close margins in individual non‐tongue subsites, and to define roles for adjuvant treatment in such cases.

## Supporting information


**Figure S1.** Kaplan Meier curve for local recurrence according to millimeter of margin clearance among patients with tongue (A) and non‐tongue (B) SCC.

Tables S1–S4.

## Data Availability

Data available on reasonable request from authors.

## References

[1] M. R. Buchakjian , T. Ginader , K. K. Tasche , N. A. Pagedar , B. J. Smith , and S. M. Sperry , “Independent Predictors of Prognosis Based on Oral Cavity Squamous Cell Carcinoma Surgical Margins,” Otolaryngology and Head and Neck Surgery 159, no. 4 (2018): 675–682, 10.1177/0194599818773070. PMC6341475 29737907

[2] D. Brinkman , D. Callanan , H. Jawad , et al., “Comparison of Royal College of Pathologists and College of American Pathologists Definition for Positive Margins in Oral Cavity Squamous Cell Carcinoma,” Oral Oncology 127 (2022): 105797, 10.1016/j.oraloncology.2022.105797. 35272227

[3] M. S. Bajwa , D. Houghton , K. Java , et al., “The Relevance of Surgical Margins in Clinically Early Oral Squamous Cell Carcinoma,” Oral Oncology 110 (2020): 104913, 10.1016/j.oraloncology.2020.104913. 32711167

[4] J. Bernier , J. S. Cooper , T. F. Pajak , et al., “Defining Risk Levels in Locally Advanced Head and Neck Cancers: A Comparative Analysis of Concurrent Postoperative Radiation Plus Chemotherapy Trials of the EORTC (#22931) and RTOG (# 9501),” Head & Neck 27, no. 10 (2005): 843–850, 10.1002/hed.20279. 16161069

[5] E. Fridman , S. Na'ara , J. Agarwal , et al., “The Role of Adjuvant Treatment in Early‐Stage Oral Cavity Squamous Cell Carcinoma: An International Collaborative Study,” Cancer 124, no. 14 (2018): 2948–2955, 10.1002/cncr.31531. 29757457 PMC6607430

[6] C. R. Anderson , K. Sisson , and M. Moncrieff , “A Meta‐Analysis of Margin Size and Local Recurrence in Oral Squamous Cell Carcinoma,” Oral Oncology 51, no. 5 (2015): 464–469, 10.1016/j.oraloncology.2015.01.015. 25716108

[7] K. K. Tasche , M. R. Buchakjian , N. A. Pagedar , and S. M. Sperry , “Definition of "Close Margin" in Oral Cancer Surgery and Association of Margin Distance With Local Recurrence Rate,” JAMA Otolaryngology. Head & Neck Surgery 143, no. 12 (2017): 1166–1172, 10.1001/jamaoto.2017.0548. 28445581 PMC5824301

[8] D. K. Zanoni , J. C. Migliacci , B. Xu , et al., “A Proposal to Redefine Close Surgical Margins in Squamous Cell Carcinoma of the Oral Tongue,” JAMA Otolaryngology. Head & Neck Surgery 143, no. 6 (2017): 555–560, 10.1001/jamaoto.2016.4238. 28278337 PMC5473778

[9] L. S. Wong , J. McMahon , J. Devine , et al., “Influence of Close Resection Margins on Local Recurrence and Disease‐Specific Survival in Oral and Oropharyngeal Carcinoma,” British Journal of Oral & Maxillofacial Surgery 50, no. 2 (2012): 102–108, 10.1016/j.bjoms.2011.05.008. 21742422

[10] S. Ch'ng , S. Corbett‐Burns , N. Stanton , et al., “Close Margin Alone Does Not Warrant Postoperative Adjuvant Radiotherapy in Oral Squamous Cell Carcinoma,” Cancer 119, no. 13 (2013): 2427–2437, 10.1002/cncr.28081. 23576156

[11] E. A. Dik , S. M. Willems , N. A. Ipenburg , S. O. Adriaansens , A. J. Rosenberg , and R. J. van Es , “Resection of Early Oral Squamous Cell Carcinoma With Positive or Close Margins: Relevance of Adjuvant Treatment in Relation to Local Recurrence: Margins of 3 Mm as Safe as 5 Mm,” Oral Oncology 50, no. 6 (2014): 611–615, 10.1016/j.oraloncology.2014.02.014. 24630900

[12] B. K. Welinder , M. Lawaetz , L. M. Dines , and P. Homøe , “No Difference in Disease‐Free Survival After Oral Cancer Resection With Close Tumor Margins in Patients With and Without Postoperative Radiotherapy,” Ear, Nose, & Throat Journal 97, no. 9 (2018): 314–322, 10.1177/014556131809700921. 30273431

[13] M. M. Justesen , H. Stampe , K. K. Jakobsen , et al., “Impact of Tumor Subsite on Survival Outcomes in Oral Squamous Cell Carcinoma: A Retrospective Cohort Study From 2000 to 2019,” Oral Oncology 149 (2024): 106684, 10.1016/j.oraloncology.2024.106684. 38211527

[14] M. Alicandri‐Ciufelli , M. Bonali , A. Piccinini , et al., “Surgical Margins in Head and Neck Squamous Cell Carcinoma: What Is 'close'?,” European Archives of Oto‐Rhino‐Laryngology 270, no. 10 (2013): 2603–2609, 10.1007/s00405-012-2317-8. 23271033

[15] L. Calabrese , M. E. Bizzoca , R. Grigolato , et al., “From Bench to Bedside in Tongue Muscle Cancer Invasion and Back Again: Gross Anatomy, Microanatomy, Surgical Treatments and Basic Research,” Life (Basel) 10, no. 9 (2020): 197, 10.3390/life10090197. 32932638 PMC7554763

[16] S. Mohamed , H. Jawad , R. O. Sullivan , D. Callanan , P. Sheahan , and L. Feeley , “Significance of Worst Pattern of Invasion‐5 in Early‐Stage Oral Cavity Squamous Cell Carcinoma,” Head and Neck Pathology 17, no. 3 (2023): 679–687, 10.1007/s12105-023-01571-9. 37486537 PMC10513981

[17] D. Brinkman , D. Callanan , R. O'Shea , H. Jawad , L. Feeley , and P. Sheahan , “Impact of 3 Mm Margin on Risk of Recurrence and Survival in Oral Cancer,” Oral Oncology 110 (2020): 104883, 10.1016/j.oraloncology.2020.104883. 32659737

[18] A. Binahmed , R. W. Nason , and A. A. Abdoh , “The Clinical Significance of the Positive Surgical Margin in Oral Cancer,” Oral Oncology 43, no. 8 (2007): 780–784, 10.1016/j.oraloncology.2006.10.001. 17174145

[19] K. Young , H. Bulosan , C. C. Kida , A. F. Bewley , M. Abouyared , and A. C. Birkeland , “Stratification of Surgical Margin Distances by the Millimeter on Local Recurrence in Oral Cavity Cancer: A Systematic Review and Meta‐Analysis,” Head & Neck 45, no. 5 (2023): 1305–1314, 10.1002/hed.27339. 36891759 PMC10079646

[20] M. Otsuru , T. Hasegawa , N. Yamakawa , et al., “A Multicenter Study on the Effect of Margin Distance on Survival and Local Control in Stage 1‐2 Squamous Cell Carcinoma of the Tongue,” Annals of Surgical Oncology 30, no. 2 (2023): 1158–1166, 10.1245/s10434-022-12462-8. 36125567

[21] C. P. Barry , F. Ahmed , S. N. Rogers , et al., “Influence of Surgical Margins on Local Recurrence in T1/T2 Oral Squamous Cell Carcinoma,” Head & Neck 37, no. 8 (2015): 1176–1180, 10.1002/hed.23729. 24798182

[22] M. R. Buchakjian , K. K. Tasche , R. A. Robinson , N. A. Pagedar , and S. M. Sperry , “Association of Main Specimen and Tumor bed Margin Status With Local Recurrence and Survival in Oral Cancer Surgery,” JAMA Otolaryngology. Head & Neck Surgery 142, no. 12 (2016): 1191–1198, 10.1001/jamaoto.2016.2329. 27423460

[23] R. S. Patel , D. P. Goldstein , J. Guillemaud , et al., “Impact of Positive Frozen Section Microscopic Tumor Cut‐Through Revised to Negative on Oral Carcinoma Control and Survival Rates,” Head & Neck 32, no. 11 (2010): 1444–1451, 10.1002/hed.21334. 20091833

[24] M. G. Bulbul , O. Tarabichi , R. K. Sethi , A. S. Parikh , and M. A. Varvares , “Does Clearance of Positive Margins Improve Local Control in Oral Cavity Cancer? A Meta‐Analysis,” Otolaryngology and Head and Neck Surgery 161, no. 2 (2019): 235–244, 10.1177/0194599819839006. 30912991

[25] J. Y. Jang , N. Choi , Y. H. Ko , et al., “Differential Impact of Close Surgical Margin on Local Recurrence According to Primary Tumor Size in Oral Squamous Cell Carcinoma,” Annals of Surgical Oncology 24, no. 6 (2017): 1698–1706, 10.1245/s10434-016-5497-4. 27519352

[26] A. Singh , A. Mishra , H. Singhvi , et al., “Optimum Surgical Margins in Squamous Cell Carcinoma of the Oral Tongue: Is the Current Definition Adequate?,” Oral Oncology 111 (2020): 104938, 10.1016/j.oraloncology.2020.104938. 32739791

[27] A. Mishra , A. Malik , S. Datta , et al., “Defining Optimum Surgical Margins in Buccoalveolar Squamous Cell Carcinoma,” European Journal of Surgical Oncology 45, no. 6 (2019): 1033–1038, 10.1016/j.ejso.2019.01.224. 30777600

[28] W. Y. Chiou , H. Y. Lin , F. C. Hsu , et al., “Buccal Mucosa Carcinoma: Surgical Margin Less Than 3 Mm, Not 5 Mm, Predicts Locoregional Recurrence,” Radiation Oncology 5 (2010): 79, 10.1186/1748-717X-5-79. 20840791 PMC2946296

[29] J. H. Maxwell , L. D. Thompson , M. S. Brandwein‐Gensler , et al., “Early Oral Tongue Squamous Cell Carcinoma: Sampling of Margins From Tumor Bed and Worse Local Control,” JAMA Otolaryngology. Head & Neck Surgery 141, no. 12 (2015): 1104–1110, 10.1001/jamaoto.2015.1351. 26225798 PMC5242089

[30] M. Amit , S. Na'ara , L. Leider‐Trejo , et al., “Improving the Rate of Negative Margins After Surgery for Oral Cavity Squamous Cell Carcinoma: A Prospective Randomized Controlled Study,” Head & Neck 38, no. Suppl 1 (2016): E1803–E1809, 10.1002/hed.24320. 26685937

[31] S. S. Wu , N. Woody , J. Hesse , et al., “Margin Assessment Methods in Oral Cavity Squamous Cell Carcinoma and Recurrence: Tumor Bed vs Resection Specimen Sampling,” JAMA Otolaryngology. Head & Neck Surgery 149, no. 11 (2023): 1011–1020, 10.1001/jamaoto.2023.2982. 37768650 PMC10540056

