# Supplementary figures and images for: Impact of Close Margins on Oral Cancer Outcomes According to the Oral Subsite
Source: Head Neck. 2024 Dec 9;47(4):1176–84. doi: 10.1002/hed.28024 (PMC11907674; doi:10.1002/hed.28024)

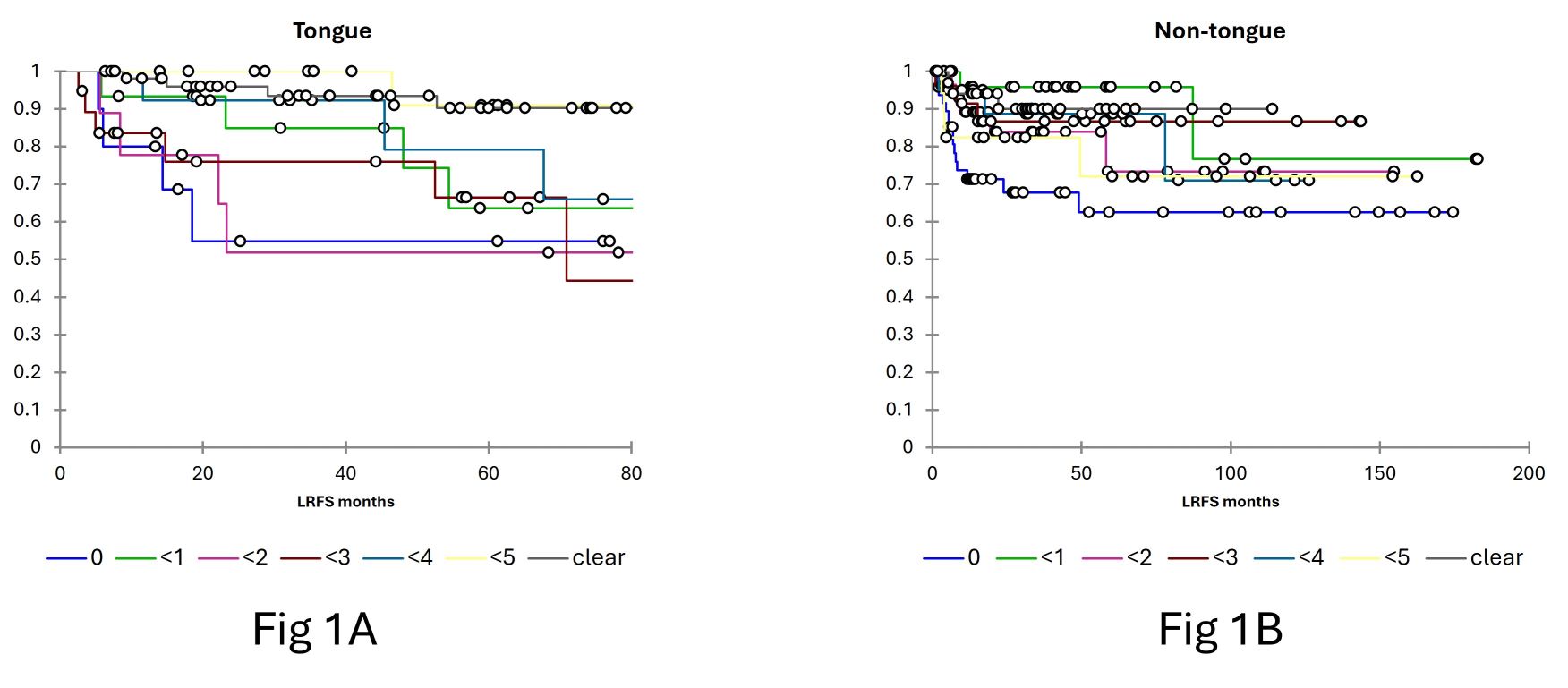

Supplement: Supplementary file 1 — Figure S1. Kaplan Meier curve for local recurrence according to millimeter of margin clearance among patients with tongue (A) and non‐tongue (B) SCC. [file HED-47-1176-s001.jpg]
