# Supplementary material for: Impact of Close Margins on Oral Cancer Outcomes According to the Oral Subsite
Source: Head Neck. 2024 Dec 9;47(4):1176–84. doi: 10.1002/hed.28024 (PMC11907674; doi:10.1002/hed.28024)
Supplement: Supplementary file 2 — Tables S1–S4. [file HED-47-1176-s002.docx]

|  |  | Univariate hazard ratio (95% CI) | p-value | Multivariate hazard ratio (95% CI) | p-value |
| --- | --- | --- | --- | --- | --- |
| Non-cohesive invasive front |  | 2.01 (0.92, 4.41) | 0.08 | Not entered |  |
| Perineural invasion |  | 3.20 (1.48, 6.91) | 0.003 | 1.67 (0.68, 4.54) | 0.27 |
| Lymphovascular invasion |  | 2.10 (0.92, 4.80) | 0.08 | Not entered |  |
| T3/4 classification |  | 3.69 (1.72, 7.90) | 0.001 | 1.44 (0.52, 3.99) | 0.48 |
| pN+ status |  | 3.72 (1.74, 7.97) | 0.001 | 1.46 (0.50, 4.25) | 0.49 |
| Extranodal extension |  | 6.88 (3.05, 15.55) | <0.0001 | 2.65 (0.78, 9.01) | 0.12 |
| Margins | Involved | 2.82 (0.73, 10.95) | 0.13 | 1.14 (0.23, 5.63) | 0.87 |
|  | Close | 1.81 (0.75, 4.36) | 0.19 | 1.19 (0.45, 3.14) | 0.73 |
| Postoperative radiotherapy |  | 3.57 (1.51, 8.47) | 0.004 | 1.45 (0.47, 4.54) | 0.52 |

Supplementary Table 1: Univariate and multivariate analysis of factors associated with DSS in Tongue SCC

|  |  | Univariate hazard ratio (95% CI) | p-value | Multivariate hazard ratio (95% CI) | p-value |
| --- | --- | --- | --- | --- | --- |
| Non-cohesive invasive front |  | 1.90 (1.10, 3.26) | 0.02 | 1.03 (0.51, 2.07) | 0.93 |
| Perineural invasion |  | 2.25 (1.32, 3.82) | 0.003 | 1.42 (0.69, 2.91) | 0.39 |
| Lymphovascular invasion |  | 1.77 (0.96, 3.25) | 0.07 | Not entered |  |
| T3/4 classification |  | 3.38 (1.98, 5.77) | <0.0001 | 2.80 (1.30, 6.03) | **0.008** |
| pN+ status |  | 3.08 (1.81, 5.25) | <0.0001 | 2.42 (1.19, 4.92) | **0.01** |
| Extranodal extension |  | 4.60 (2.39, 8.87) | <0.0001 | 1.46 (0.61, 3.53) | 0.40 |
| Margins | Involved | 1.99 (0.66, 6.03) | 0.22 | 1.28 (0.67, 4.31) | 0.78 |
|  | Close | 1.81 (0.99, 3.30) | 0.05 | 1.27 (0.66, 2.45) | 0.46 |
| Postoperative radiotherapy |  | 1.59 (0.94, 2.70) | 0.09 | 0.47 (0.23, 0.97) | **0.04** |

Supplementary Table 2: Univariate and multivariate analysis of factors associated with OS in Tongue SCC

|  |  | Univariate hazard ratio (95% CI) | p-value | Multivariate hazard ratio (95% CI) | p-value |
| --- | --- | --- | --- | --- | --- |
| Non-cohesive invasive front |  | 1.73 (0.99, 3.01) | 0.05 | 0.79 (0.40, 1.55) | 0.49 |
| Perineural invasion |  | 3.19 (1.83, 5.55) | <0.0001 | 2.52 (1.29, 4.93) | **0.007** |
| Lymphovascular invasion |  | 3.12 (1.64, 5.92) | 0.001 | 1.89 (0.91, 3.93) | 0.09 |
| T3/4 classification |  | 4.58 (2.39, 8.79) | <0.0001 | 2.97 (1.36, 5.88) | **0.005** |
| pN+ status |  | 4.06 (2.29, 7.22) | <0.0001 | 2.98 (1.42, 6.18) | **0.004** |
| Extranodal extension |  | 3.59 (2.01, 6.40) | <0.0001 | 2.08 (0.97, 4.44) | 0.06 |
| Margins | Involved | 2.62 (1.05, 6.56) | 0.04 | 4.83 (1.76, 13.24) | **0.001** |
|  | Close | 1.12 (0.46, 2.75) | 0.80 | 1.68 (0.67, 4.23) | 0.27 |
| Postoperative radiotherapy |  | 0.84 (0.49, 1.46) | 0.54 | 0.26 (0.14, 0.49) | **<0.0001** |

Supplementary Table 3: Univariate and multivariate analysis of factors associated with DSS in Non-Tongue SCC

|  |  | Univariate hazard ratio (95% CI) | p-value | Multivariate hazard ratio (95% CI) | p-value |
| --- | --- | --- | --- | --- | --- |
| Non-cohesive invasive front |  | 1.15 (0.80, 1.63) | 0.46 | Not entered |  |
| Perineural invasion |  | 2.03 (1.38, 2.97) | 0.0002 | 1.99 (1.32, 3.02) | **0.001** |
| Lymphovascular invasion |  | 2.29 (3.74, 1.40) | 0.001 | 1.82 (1.03, 3.19) | **0.04** |
| T3/4 classification |  | 1.72 (1.20, 2.46) | 0.003 | 1.51 (0.8 2.33) | 0.06 |
| pN+ status |  | 2.07 (1.44, 2.98) | <0.0001 | 2.30 (1.33, 3.97) | **0.003** |
| Extranodal extension |  | 2.24 (1.46, 3.43) | 0.0002 | 1.54 (0.87, 2.73) | 0.14 |
| Margins | Involved | 1.08 (0.61, 1.89) | 0.63 | 1.69 (0.92, 3.12) | 0.09 |
|  | Close | 0.79 (0.48, 1.31) | 0.37 | 1.12 (0.66, 1.88) | 0.68 |
| Postoperative radiotherapy |  | 0.56 (0.39, 0.80) | 0.001 | 0.26 (0.18, 0.40) | **<0.0001** |

Supplementary Table 4: Univariate and multivariate analysis of factors associated with OS in Non-Tongue SCC
